# Denial and Empathy: Partners in Employee Trust Repair?

**DOI:** 10.3389/fpsyg.2019.00019

**Published:** 2019-01-22

**Authors:** Zhanna Bagdasarov, Shane Connelly, James F. Johnson

**Affiliations:** ^1^Department of Management, California State University, Fresno, Fresno, CA, United States; ^2^Department of Psychology, The University of Oklahoma, Norman, OK, United States; ^3^Strategic Research and Assessment Branch, Air Force Personnel Center, Randolph Air Force Base, TX, United States

**Keywords:** trust repair, empathic ability, integrity violation, affect, response to violation

## Abstract

Prior research on trust repair has focused primarily on investigating verbal responses to breaches of trust. Although consistently implicated in violations, the role of affect in the repair process has been mostly ignored. Using a scenario-based paradigm, we conducted an experimental study to examine the value of mistrusted party’s empathy, specific responses to an integrity-based violation (apology vs. denial), and nature of consequences (personal vs. organizational), as well as their interactive effects, on trust repair. Consequently, we sought to merge work on verbal responses with affect. Major findings indicated that presence of mistrusted party’s empathy functioned to repair trust better than its absence and, when coupled with a denial of culpability, produced markedly increased perceptions of violator’s integrity. These findings contribute to our understanding of how leaders influence followers through affect, informing both emotion and trust theory.

## Introduction

Literature is replete with evidence showing that interpersonal trust in organizational settings has direct and indirect effects on key work outcomes such as individual and team performance, organizational commitment, turnover intentions, job satisfaction, and organizational citizenship behaviors (Deluga, 1995; Kramer, 1999; Flaherty and Pappas, 2000; Dirks and Ferrin, 2001, 2002; Breuer et al., 2016; De Jong et al., 2016). Organizational transgressions damage interpersonal trust (Lewicki and Wiethoff, 2000). This is problematic because organizational misconduct is widespread, with over 50% of employees experiencing a breach of trust within their organizations (Robinson and Rousseau, 1994; Guest and Conway, 2002; Schweitzer et al., 2006). Additionally, trust violations have an enduring impact on those harmed, with 50% of employees recalling workplace transgressions for up to 20 years (Jones and Burdette, 1994). The damage to interpersonal trust can have devastating effects on various organizationally relevant outcomes. For instance, broken interpersonal trust may lessen cooperative behavior between parties involved in the trust violation, and increase revenge seeking (Dirks et al., 2009). When trust in organizations is violated, customer satisfaction and loyalty are also reduced, ultimately affecting company profits (Leonidou et al., 2013).

Fortunately, research demonstrates that broken trust *can* be restored (Mishra, 1996; Bottom et al., 2002), but it can be contingent on the violator’s response to the accusation (Kim et al., 2004, 2006; Schweitzer et al., 2006; Ferrin et al., 2007). The bulk of this work has examined the effects of these responses based on the nature of the misconduct (e.g., integrity-based or competence-based misconduct), but not based on the nature of the consequences of the misconduct (i.e., whether the consequences were of a personal or organizational nature). Furthermore, the role of affect—in contrast to cognition—in trust repair has largely been ignored (Kramer and Lewicki, 2010; Chen et al., 2011; Lockey, 2017; Mölders et al., 2017). We aim to fill these gaps by further differentiating trust violations in terms of personal and organizational consequences and by examining the influence of affective response. Most importantly, this work examines *how* responses can be given to render them more effective. In fact, in their recent review of trust repair literature, Lewicki and Brinsfield (2017) specified that it is currently not clear how some verbal responses, specifically denial, should be combined with other repair strategies, such as utilization of empathy. Trust repair research has not yet delved into the affective elements of a response that might make these responses appear more sincere. We address this particular comment by demonstrating one way for coupling denial with displays of empathy to facilitate trust repair efforts.

### Trust, Violations, and Responses

Trust is commonly defined as a willingness to accept vulnerability based on positive expectations regarding the behavior of another (Mayer et al., 1995; Rousseau et al., 1998). This definition further involves two elements: *trusting intentions* and *trusting beliefs*. Trusting intentions refer to the willingness to make oneself vulnerable to another in the presence of risk (denoted as *willingness to risk* in our study), while trusting beliefs refer to the beliefs about another’s integrity that may lead to trusting intentions (designated as *perceived integrity* in our study) (McKnight et al., 1998). Both trusting beliefs and intentions must be intact in order for successful trust repair to occur (Kim et al., 2004; Tomlinson and Mayer, 2009).

Mayer et al. (1995) provide a well-cited conceptual foundation for scholarship on trust repair (Schoorman et al., 2007). Their model of organizational trust posits that a determination of trustworthiness is based on an assessment of the potential trustee’s ability, benevolence, and integrity. Ability is the extent to which trustees possess skills and competencies allowing them to contribute to the trustor’s well-being (Mayer et al., 1995). Benevolence refers to a trustee’s desire to bestow goodwill onto the trustor. Integrity is the extent to which the trustee adheres to a set of principles that the trustor finds acceptable. If trust is somehow damaged, the three characteristics, or some combination of them, will be reevaluated (Mayer et al., 1995). Thus, damaged trust provides impetus for recalibration of the trustworthiness of a trustee. Additionally, because trustworthiness precedes trust, trust can be repaired by increasing the specific dimension of trustworthiness that suffered a decline (Tomlinson and Mayer, 2009).

#### Response to the Violation

Numerous studies have investigated the effects of violators’ responses following a transgression, with the majority focusing on the effect of only the mistrusted party’s response (e.g., apology, denial, and reticence) on trust outcomes (i.e., trusting intentions, trusting beliefs, and hiring decisions) (Ferrin and Dirks, 2003; Kim et al., 2004, 2006; Ferrin et al., 2007; Dirks et al., 2011). Such studies have revealed that trust was more likely to be repaired when the transgressor apologized for a competence-based violation but denied involvement in the integrity-based breach (Kim et al., 2004, 2006; Ferrin et al., 2007). Additionally, Ferrin et al. (2007) examined the effect of reticence (the accused party neither confirmed nor denied the allegation) on trust repair. Reticence was shown to be a suboptimal response to an integrity violation, as was an apology. Given that trust is more likely to be repaired when the perpetrator denies involvement rather than apologizes for an integrity-based violation, we expect similar findings in our study.

Although researchers have differentiated between integrity- and competence-based violations as means of studying integrity and ability dimensions of trustworthiness, they have yet to delve deeper into either as a method. Here, we probe further into integrity-based violations, focusing on increasing the integrity dimension of perceived trustworthiness. Tsai et al. (2010), for instance, provided support for the idea that trust erodes more following integrity-based rather than competence-based violations. Others, too, have come to a similar conclusion, proposing that integrity violations are viewed as failures in moral judgment, while competence violations can be mistakes or misunderstandings (Kim et al., 2004, 2006, 2013). Consequently, we chose to investigate trust repair after most damaging types of violation. By doing so, we hope to provide recommendations for strengthening the integrity dimension of trustworthiness by means of affective displays, which has yet to be studied.

#### Nature of Consequences

The effectiveness of verbal responses in trust repair may also be affected by the nature of the consequences of integrity-based transgressions. Negative emotions such as disappointment, frustration, anger, and outrage by the harmed individual following a violation have been well-documented (Bies and Tripp, 1996; Lewicki and Bunker, 1996; Morrison and Robinson, 1997; Mikula et al., 1998; Conway and Briner, 2002; Barclay et al., 2005). However, individuals do not experience the full range of these emotions when considering the feelings of others who have had their trust violated (Rust et al., 2005). Thus, it is possible that violations carrying personal consequences (i.e., outcomes relevant to oneself) will evoke stronger reactions than organizational consequences (i.e., outcomes relevant for the entire organization than one particular person). Although hearing about a transgression affecting others would likely induce some sense of concern within an individual, being personally affected by a violation would be expected to produce more pronounced negative emotions. We therefore propose these stronger negative reactions to personal violations are likely to lead to a decrease in trust repair outcomes. While this proposition is novel within the trust literature, research in psychological contract violations suggests that the nature of consequences plays an important role in how employees react to a breach of contract. Breaches in psychological contracts, as in integrity violations, involve a failure on the part of the organization or a particular individual (Robinson and Rousseau, 1994), making the two comparable in nature. Furthermore, akin to an integrity violation, a psychological contract violation “…captures a focal person’s emotional responses including frustration and anger that follow breach” (Zhao et al., 2007, p. 650). Thus, both types of violations share similarities in how they are defined and the affective responses that follow. Rust et al. (2005), for instance, showed that employees perceived their own layoffs from an organization to be a more significant breach of contract than the layoffs of other employees. Findings further indicated that employee reactions to a personal psychological contract breach were more severe and emotional than reactions to similar violations against others. Considering these findings, we propose the following:

H_1_: Integrity-based violations resulting in personal rather than organizational consequences will result in (1a) lower perceived integrity of the violator and (1b) less willingness to risk (or make oneself vulnerable).
H_2_: The effects of denial to a trust breach will be moderated by the nature of the consequences such that denial that occurs with personal consequences of a trust breach (versus organizational consequences) will result in (2a) lower perceived integrity of the violator, and (2b) less willingness to risk.

In addition to these hypotheses, we seek to confirm the presence of negative emotional reactions in harmed parties of integrity-based trust violations and investigate the differential impact of the response given by a transgressor and the nature of the consequences on individuals’ affective reactions. Research has repeatedly shown denial to be the more successful response in repairing trust following integrity-based violations (Kim et al., 2004, 2006; Ferrin et al., 2006), perhaps indicating that apology activates a more unpleasant emotional response in people harmed by a trust violation. It could be that confirming culpability by apologizing—as opposed to leaving room for doubt about culpability by denying fault—leads to more negative feelings about the apologizer (Kim et al., 2004). Thus, we make the following predictions:

H_3_: Individuals will exhibit higher negative affect when the violator responds with an apology as opposed to denial.
H_4_: Individuals will exhibit higher negative affect when the nature of the violation is personal rather than organizational.

### Affect and Trust

Few studies have considered the role of affect in trust repair. In fact, affect has been mostly absent from models of trust (Mayer et al., 1995; Schoorman et al., 2007), and where emotions are considered, it is typically in regard to trust *development* rather than *repair*. A few researchers have begun to acknowledge the importance of affect in interpersonal trust (Jones and George, 1998; Williams, 2001, 2007; Dunn and Schweitzer, 2005; Schoorman et al., 2007; Tomlinson and Mayer, 2009; Chen et al., 2011), but none have integrated affect into a trust model. Studying incidental emotions, Dunn and Schweitzer (2005) demonstrated that positive emotional states increased trust, while negative emotional states decreased trust. Lount (2010) explored the impact of mood on trust in interpersonal interactions and found that, when in a positive mood, people’s trust in another party increased when they perceived cues associated with trust, but decreased when they perceived cues characterized by distrust. Similarly, Mislin et al. (2015) showed that individuals in a happy mood exhibited an increase in trust behavior. Though these few studies demonstrate that affect and trust are being examined in a limited way, they showcase the need for a rigorous investigation of emotions and trust in a *repair* context. While related, the dynamics of establishing trust are qualitatively different than the dynamics of repairing a pre-existing trust relationship that has been damaged.

The Emotions as Social Information model (EASI; Van Kleef, 2008a,b) provides the theoretical basis for our hypotheses regarding the role of affect in trust repair. EASI positions emotions as sources of information about an individual’s thoughts and intentions that influence the behavior of others via two potential paths: *strategic information* or *affective reactions* (Van Kleef, 2008a). Emotions conveyed as strategic information allow others to draw strategic inferences and thereby determine their behavior as reactions to the information, while emotions conveyed via the affective reactions path exert influence by eliciting affective reactions in others (Van Kleef, 2008a). Given the tenets of this model, we propose that coupling affective displays and verbal responses will increase trust repair efforts. If the transgressor exhibits an emotional display while responding to the harmed party following a transgression, EASI suggests that affective reactions are more likely to be elicited in the trustor and useful information about the trustee may be inferred. To this end, Tomlinson and Mayer (2009) proposed that something must be done to offset or reduce the negative emotions being experienced by the harmed party in order to restore trust. Reducing negative emotions will lead to more rational processing of information by lessening cognitive load (Tomlinson and Mayer, 2009). We employ a display of empathy by the transgressor as a route to trust repair and as a means to mitigate negative emotions.

#### Empathy

Empathy is defined as “the ability to comprehend another’s feelings and to re-experience them oneself” (Salovey and Mayer, 1990, p. 194). Most researchers agree that empathy involves both affective and cognitive components (Miller and Eisenberg, 1988; Duan and Hill, 1996), with many discussing empathy in terms of emotional ability, capacity, or skill (e.g., Kellett et al., 2002). While a majority of the work on empathy isolates it from emotion or emotion-related theories, Wondra and Ellsworth (2015) proposed an appraisal theory of vicarious emotional experiences, treating empathy as a normal part of emotional experience. The authors state, “[…] empathic emotions are real emotions” (Wondra and Ellsworth, 2015, p. 411), clarifying throughout their work that empathy is a vicarious emotion rather than a first-hand emotion. We recognize the role of affect in empathy and follow Wondra and Ellsworth (2015) in not isolating it from theories of emotion. Accordingly, we extend the use of EASI to our predictions regarding empathic capabilities and trust repair.

Research shows that followers react favorably to leaders who display empathy (Kellett et al., 2002, 2006; Pescosolido, 2002; Holt et al., 2017) and that empathy can help facilitate mutual trust (Bass, 1960; Mahsud et al., 2010). Kellett et al. (2006) found evidence for the usefulness of leader empathy in interpersonal relationships. Similarly, a study by Thiel et al. (2013) suggested that leader empathy may be important in attenuating negative emotions and defusing stress associated with a state of crisis. More recently, Young et al. (2017) demonstrated that participants exposed to leader empathic concern while receiving negative feedback experienced a decline in negative affect and an increase in positive affect. They were also likely to evaluate the leader’s behavior as more effective than their control counterparts. Though we could find no previous work investigating the role of empathy in trust repair following integrity breaches, Nadler and Liviatan (2006) established the usefulness of empathy in reconciliation efforts. The authors showed that an out-group’s expression of empathy for an in-group’s suffering resulted in increased willingness to reconcile on the part of the in-group. This finding, however, was only true in cases where trust between parties was high. When trust was low, an opposite effect was observed. These studies and relevant others all indicate that empathy can function to regulate others’ negative emotions, boosting positive affect and enhancing interpersonal relationships. This idea supports a large body of research demonstrating that agents who regulate a target’s emotions enjoy higher perceptions of trust and friendship (Niven et al., 2012; Troth et al., 2018).

The clinical and counseling literature provide additional support for our proposition that empathy may aid in trust repair. Barrett-Lennard (1981, p. 94) characterized received empathy as having a “healing and growth-enhancing” effect, suggesting the value of empathy in mitigating negative affect. Additionally, Bachelor (1988) content-analyzed written work by people undergoing therapy for their perceptions of therapist empathy and its resultant effects on the clients. This work produced evidence that therapist’s empathy contributed to distress-reduction in clients—with clients citing consolation, relief, alleviation of problems, appeasement, deepening of bond, acceptance, feelings of comforting warmth, consideration, and support as results of receiving empathy. Most important to the present work, Bachelor (1988, p. 236) specified that clients also cited “Increased trust and gratitude toward the therapist…” as a result of empathy. These studies suggest that empathy allows the receiver to experience relief and positive affect as a result of feeling understood, which may translate into facilitating trust repair. Given these findings, we propose:

H_5_: If the violator displays empathy rather than no empathy, individuals will (5a) perceive the violator as having higher integrity, (5b) exhibit higher willingness to risk, and exhibit lower (5c) negative affect.
H_6_: Response to the violation (i.e., apology vs. denial) and violator empathy will interact such that presence of denial and empathy will result in (6a) higher perceived integrity of the violator, and (6b) willingness to risk, and (6c) lower negative affect.
H_7_: Violator empathy and nature of consequences will interact to predict increased (7a) perceived integrity of the violator and (7b) willingness to risk when an organizational violation is combined with violator empathy, and increased (7c) negative affect when a personal violation is coupled with no violator empathy.

## Materials and Methods

### Participants

Two hundred and forty undergraduate students from a large, public university in the Southwestern United States took part in this study. Participants were recruited via an online experiment management system and granted course credit in exchange for participation. Participation, however, was done in person. On average, participants were 19.28 years old (*SD* = 1.41; range = 18–32 years). Of these participants, 29.6% were male, 66.7% were female, and 3.8% chose not to report their gender. Furthermore, participants in this sample possessed an average of 2.4 years of work experience (*SD* = 1.96), with the majority of the sample lacking any face-to-face (80.4%) or online (72.8%) ethics or integrity training.

### Design and Procedure

Participants were randomly assigned to one of eight conditions in a 2 (nature of integrity violation: personal vs. organizational consequences) × 2 (response to violation: apology vs. denial) × 2 (violator empathy: present vs. absent) between-subjects factorial design.

Participants took part in this study in groups of approximately 10 individuals. The number of participants in a given session depended upon the number of individuals signed up for a particular time slot. Study administrators began by distributing and audibly reviewing the informed consent form, allowing participants ample time to read and understand the nature of the study. Participants were asked to sign the consent form if they wished to participate.

All study materials were provided in two packets. First, participants received a packet containing the vignette with manipulated content corresponding to the condition the individual was assigned to. This packet concluded with open- and closed-ended questions assessing participants’ affective reaction to the case content and post-manipulation questionnaires assessing trust outcomes. After completing and submitting the first packet, participants received the second packet. This packet contained a covariate measure of trust and cynicism, a brief demographic survey, and several questions used to check whether the manipulations had the intended effect.

### Study Case and Manipulations

All manipulations were embedded within a vignette by varying the content of the same scenario according to the conditions. Participants read a one-page scenario asking them to assume the role of an accountant within a large accounting department at a start-up electronics company.

#### Nature of the Violation

The nature of the violation refers to and involves either a personal or an organizational consequence. The same scenario was manipulated to include descriptions of very personal outcomes for the participant or organizational consequences. Personal outcomes involved the main character being passed over for a well-deserved promotion, while organizational consequences entailed the entire accounting firm losing their annual bonuses, both as a result of an integrity-based violation committed by the boss.

#### Response to the Violation

Following a description of the setting and the perpetration of an integrity-based violation by the boss, the scenario features the main character (i.e., participant) directly confronting the boss about the violation. The transgressor responds to this confrontation with either an apology or a denial. As in previous studies examining verbal responses (Kim et al., 2004, 2013), the transgressor admits responsibility when apologizing or completely denies culpability for the violation.

#### Violator Empathy

Along with the verbal responses to the trust violation, the transgressor’s speech was manipulated to either contain or be devoid of empathy. Empathy was portrayed via the content and tone of the perpetrator’s response to being accused. When a response contained empathy, the violator demonstrated understanding of participant’s anger, while communicating a general tone of compassion and concern.

Please see Appendix A for samples of a manipulated scenarios.

### Dependent Variables

We evaluated participants’ trust in their boss (i.e., transgressor) using several frequently utilized measures in the trust repair literature. We adapted a commonly used measure of trusting beliefs (i.e., perceived integrity), and trusting intentions (i.e., willingness to risk) to include the name of the transgressor (i.e., Chris Johnson) in order to make these scales more relevant to the current study. Otherwise, the scales remained unaltered from those used by Kim et al. (2004).

#### Trust Outcomes

##### Perceived integrity

Participants’ trusting beliefs were assessed by their perceptions of the violator’s integrity following the transgression (Mayer and Davis, 1999). This adapted 3-item scale asked participants to rate on a 7-point Likert scale the extent to which they agreed or disagreed with each statement. A sample item included: “Sound principles seem to guide Chris Johnson’s behavior.” Cronbach’s alpha for this scale was 0.89, indicating a high degree of internal consistency among the three items. Please see Appendix B for the full questionnaire.

##### Willingness to risk

Participants’ intentions to trust the violator were measured by an adapted version of the Mayer and Davis (1999) “willingness to risk” scale. This measure was comprised of three items. A sample item stated: “I wouldn’t let Chris Johnson have any influence over issues that are important to me.” The items were measured on a 7-point Likert scale (1 = *strongly disagree*, 7 = *strongly agree*), with two items requiring reverse scoring. Cronbach’s alpha for this scale was 0.67. This relatively low internal consistency coefficient is likely related to the scale containing only three items. Please see Appendix C for the full questionnaire.

#### Affect Outcome

##### Negative affect

Participants were asked to provide an answer to two open-ended questions designed to assess their overall affective reaction to the case. Participants were asked to (1) describe their feelings about their boss’s actions, and (2) describe their feelings regarding the confrontation they just had with their boss about his actions. Responses were appraised for presence of negative affect by four trained raters. Negative affect was defined as a general dimension of subjective distress involving a variety of negative emotions, including anger, contempt, disgust, guilt, fear, and nervousness. Participants’ responses to both questions in tandem were coded for presence of negative affective tone on a five-point Likert scale (*1 = minimal or lack of negative tone*, 5 = *high negative tone*). Intraclass correlation coefficient (ICC) was used to assess inter-rater reliability. The resultant reliability coefficient was high (ICC = 0.80). Please see Appendix D for the two questions as they were presented to the participants.

#### Coding Procedures

Four coders, all of whom were blind to the conditions and hypotheses of this study, were tasked with rating the responses for presence of negative affect in the two open-ended questions. Coders received a thorough, 3-h frame-of-reference training (Bernardin and Buckley, 1981) prior to scoring any responses. Training consisted of providing operational definitions along with benchmarks for each variable and practicing rating on a randomly selected pool of responses. A follow-up meeting for calibration purposes was held 1 week after the initial training meeting. Raters were then given all participant responses and allowed to begin coding.

### Control Variables

#### Philosophies of Human Nature Scale (PHN)

A revised form of the Philosophies of Human Nature (PHN; Wrightsman, 1964, 1974) scale was used to derive two covariate variables: trust and cynicism. This 20-item scale was measured using a 7-point Likert scale (*1 = strongly disagree*, 7 = *strongly agree*), with ten items of the scale dedicated to forming each of the two variables. Participants were asked to rate the extent to which they agreed or disagreed with a number of statements about people’s general behavior. A sample trust item included: “Most people will speak out for what they believe in.” A sample item from the cynicism scale included: “Most people will tell a lie if they could gain by it.” Both cynicism and trust scales indicated good internal consistency (Cronbach’s alpha = 0.79 and 0.76, respectively).

#### Demographic Survey

The demographic survey asked participants to report their age, gender, and years of work experience. Age has been used as a covariate in studies involving the trust construct because people of diverse ages may react differently to situations concerning trust violations based on life and work experiences (Tomlinson et al., 2004).

#### Engagement in Study

As in most laboratory studies, participants’ lack of motivation to perform and follow the study rules is a concern (Goldberg and Grandey, 2007). In order to account for this potentiality, we asked participants to report their level of engagement in the study activities on a 7-point Likert scale. Cronbach’s alpha for this brief scale was 0.82, indicating a high degree of internal consistency among the three items.

### Manipulation Checks

In order to assess whether the three manipulations (i.e., nature of integrity violation, response to violation, and violator empathy) had the intended effect, three manipulation check surveys were administered within the second packet. Each survey assessed whether participants recognized the nature of the violation as either personal or organizational, the response to the violation as either apologetic or full of denial, and the affect associated with the violator’s response.

#### Nature of the Violation

Four questions, rated on a 5-point Likert scale (1 = *not at all*, 5 = *very much*), were created to assess the nature of integrity violation manipulation. Two questions were designated to evaluate the personal nature of the violation and two questions were created to assess the organizational nature of the violation. Participants were asked to rate the extent to which the ethical issue in the scenario resulted in a personal versus organizational violation and the extent to which they felt personally affected by their boss’s action versus that the entire organization was affected by the boss’s actions. Each set of two questions was averaged to create one score for each type of violation.

#### Response to the Violation

Two questions were developed to gauge the intended influence of the response to the violation manipulation. One question asked participants to rate the extent to which their boss took responsibility for the ethical issue in the scenario (apology manipulation), while the second question asked participants to rate the extent to which their boss denied responsibility for the problem in the scenario (denial manipulation). Both questions were rated on a 5-point Likert scale (1 = *not at all*, 5 = *very much*).

#### Violator Empathy

The intended effect of the violator empathy manipulation was assessed using three questions. Once again, participants were asked to rate on a 5-point scale (1 = *not at all*, 5 = *very much*) the extent to which their boss (1) appeared to care about and understand their feelings regarding the situation, (2) was empathetic, and (3) was indifferent about their feelings regarding the situation. The last question was reverse coded. These questions were ultimately averaged to create one score for the empathy manipulation.

## Results

Descriptive statistics and intercorrelations among all dependent study variables and covariates are provided in Table 1. Table 2 contains trust and affect variable means and standard deviations broken down by condition.

**Table 1:** Means, standard deviations, and intercorrelations among all covariates and dependent variables.

Variable	*M*	*SD*	1	2	3	4	5	6
(1) Perceived integrity	2.02	1.33	–					
(2) Willingness to risk	2.19	1.19	0.56^∗∗^	–				
(3) Negative affect	3.60	0.54	-0.26^∗∗^	-0.24^∗∗^	–			
**Covariates**								
(4) Age	19.28	1.41	0.01	-0.11	0.02	–		
(5) Cynicism	4.28	0.88	0.10	-0.10	0.09	-0.15^∗^	–	
(6) Trust via PHN	4.05	0.76	0.03	0.14^∗^	-0.13^∗^	0.03	0.32^∗∗^	–


*^∗^Correlations are significant at p < 0.05. ^∗∗^Correlations are significant at p < 0.01.*

**Table 2:** Means and standard deviations of trust and affect dependent variables by condition.

			Trust outcomes				Affect outcome	
				
Leader empathy	Nature of violation	Response to violation	Perceived integrity		Willingness to risk		Negative affect	
					
			*M*	*SD*	*M*	*SD*	*M*	*SD*
Absent	Personal	Apology	1.87	1.37	1.98	1.25	3.80	0.48
		Denial	1.31	0.44	2.00	0.91	3.91	0.51
	Org.	Apology	2.20	1.14	2.57	1.40	3.72	0.37
		Denial	1.63	1.16	2.03	0.96	3.80	0.54
Present	Personal	Apology	2.03	1.15	1.71	0.65	3.73	0.41
		Denial	3.00	1.80	3.00	1.52	3.10	0.55
	Org.	Apology	1.84	1.38	2.17	1.22	3.40	0.34
		Denial	2.33	1.28	2.13	1.06	3.25	0.50


*N = 240. Not adjusted for covariates.*

### Manipulation Checks

#### Nature of the Violation

Independent samples *t*-tests were conducted to test whether participants recognized the intended personal- and organizational-level manipulations. Analyses on questions aimed to assess a personal-level violation indicated that participants in the personal violation condition scored significantly higher (*M* = 4.18, *SD* = 0.81) than those in the organizational-level violation condition (*M* = 3.80, *SD* = 0.81), *t*(238) = 3.63, *p* < 0.001. For questions designed to measure the effect of the organizational-level manipulation, analyses indicated that participants exposed to the organizational manipulation scored significantly higher (*M* = 4.17, *SD* = 0.97), than those subjected to the personal-level manipulation (*M* = 3.10, *SD* = 1.20), *t*(238) = 7.62, *p* < 0.001. Both analyses suggest that the nature of the violation manipulation (both personal and organizational) had the intended impact.

#### Response to the Violation

On the question designed to evaluate the apologetic response from the boss, “To what extent did your boss take responsibility for the ethical issue in the scenario?” participants in the apology condition indicated a significantly higher level of agreement (*M* = 2.59, *SD* = 1.17) than participants in the denial condition (*M* = 1.36, *SD* = 0.77), *t*(238) = 9.56, *p* < 0.001. On the question, “To what extent did your boss deny responsibility for the ethical issue in the scenario?” participants in the denial condition indicated a significantly higher level of agreement (*M* = 4.24, *SD* = 1.24) than participants in the apology condition (*M* = 2.26, *SD* = 1.33), *t*(237) = 11.87, *p* < 0.001. Both analyses suggest that the response to the violation manipulation worked as anticipated.

#### Violator Empathy

Using an average of the three questions meant to measure whether the violator’s empathetic display was manipulated, an independent samples *t*-test showed that individuals who were exposed to the violator empathy condition scored significantly higher (*M* = 3.16, *SD* = 0.95) than those whose scenarios were devoid of empathy (*M* = 2.00, *SD* = 0.69), *t*(238) = 10.88, *p* < 0.001. This analysis showed that the empathy manipulation was also successful.

### Hypotheses Tests

#### Trust Outcomes

##### Perceived integrity and willingness to risk

Because both variables were positively skewed, had a floor, and were kurtotic, a commonly-used inverse transformation was conducted (i.e., 1/*x*, where *x* is the original number) to correct and improve normality of the distributions and equalize variances to meet the necessary assumptions for inferential statistics (Whelan, 2008). Thus, the transformed versions of these two dependent variables were ultimately used in the multivariate analysis of covariance (MANCOVA). However, in order to preserve the nature of the relationships between variables and reduce complexity when interpreting the data, we reported original values for the purposes of means, standard deviations, correlations, and graphing (Osborne, 2002). It is important to note that our findings regarding main effects and interactions for these two variables were exactly the same across transformed and untransformed analyses.

In order to test all hypotheses predicting impact on the perceived integrity and willingness to risk trusting outcomes, we conducted a 2 × 2 × 2 MANCOVA. Box’s test of equality of covariance matrices was non-significant (0.29), suggesting that the necessary assumption was met. Only age and cynicism were revealed as significant covariates, with age being marginally significant (*p* = 0.068). We kept both age and cynicism in the MANCOVA analysis using transformed dependent variables because both proved to be significant covariates when we conducted the same MANCOVA analysis on untransformed values. Keeping age in the model allowed us to keep everything uniform across transformed and untransformed analyses. The MANCOVA analysis revealed one significant multivariate effect of violator empathy, Wilks’ λ = 0.94, *F*(2,229) = 7.44, *p* < 0.01, $\eta_{p}^{2}$ = 0.06. Examination of follow-up univariate analyses showed that the effect of violator empathy was only significant for perceived integrity (hypothesis 5a), *F*(1,230) = 12.93, *p* < 0.001, $\eta_{p}^{2}$ = 0.05, and not for willingness to risk (hypothesis 5b). As predicted by hypothesis 5a, presence of violator’s empathic display resulted in higher levels of perceived integrity (*M* = 2.30, *SD* = 1.47) than no empathic display (*M* = 1.75, *SD* = 1.12). There were no additional significant main effects, suggesting that Hypotheses 1a and 1b were not supported.

The MANCOVA also revealed a marginally significant interaction for nature of the integrity violation × response to violation, Wilks’ λ = 0.98, *F*(2,229) = 2.95, *p* = 0.054, $\eta_{p}^{2}$ = 0.03. Follow-up univariate analyses indicated that the interaction was only significant for willingness to risk, *F*(1,230) = 5.76, *p* < 0.05, $\eta_{p}^{2}$ = 0.02. One-tailed simple effects analyses were conducted for nature of the violation at each level of response to the violation. The results of the simple effects tests indicated that when an individual was personally violated, denial worked better at repairing trust, *t*(120) = 2.81, *p* < 0.01. There was not a significant difference between apology and denial responses at the organizational level, *t*(116) = -0.70, *p* > 0.025. This pattern contradicts hypothesis 2b since we predicted that denial would work better at repairing trust at the organizational level rather than personal. Hypothesis 2a was not supported.

Further, the MANCOVA revealed that the nature of the integrity violation × violator empathy interaction was also significant, Wilks’ λ = 0.97, *F*(2,229) = 3.32, *p* < 0.05, $\eta_{p}^{2}$ = 0.03. Univariate analyses showed that the interaction was significant for both perceived integrity [*F*(1,230) = 5.83, *p* < 0.05, $\eta_{p}^{2}$ = 0.03] and willingness to risk [*F*(1,230) = 4.09, *p* < 0.05, $\eta_{p}^{2}$ = 0.02]. One-tailed simple effects analyses were conducted for nature of the violation at each level of violator empathy. The results of the simple effects tests showed that when the violation was of a personal nature, presence of violator empathy resulted in higher perceived integrity, *t*(120) = 4.13, *p* < 0.001. There was not, however, a significant difference at the personal violation level between presence and absence of violator empathy on willingness to risk, *t*(120) = 1.87, *p* > 0.025. There was also not a significant difference between presence or absence of violator empathy at the organizational level for either perceived integrity, *t*(116) = 0.88, *p* > 0.025, or willingness to risk, *t*(116) = -0.98, *p* > 0.025. These findings suggest that hypotheses 7a and 7b were not supported.

Finally, the MANCOVA also showed that the response to the violation × violator empathy interaction was significant, Wilks’ λ = 0.93, *F*(2,229) = 8.28, *p* < 0.001, $\eta_{p}^{2}$ = 0.07. Univariate analyses showed that the interaction was significant for both perceived integrity [*F*(1,230) = 16.58, *p* < 0.001, $\eta_{p}^{2}$ = 0.07] and willingness to risk [*F*(1,230) = 5.22, *p* < 0.05, $\eta_{p}^{2}$ = 0.02]. One-tailed simple effects analyses were conducted for nature of the violation at each level of violator empathy. Findings revealed that when the violator responded with denial, presence of violator empathy resulted in higher perceived integrity than its absence, *t*(116) = 5.60, *p* < 0.001. There was also no significant difference between presence or absence of violator empathy when the violator employed an apologetic response for either perceived integrity, *t*(120) = -0.35, *p* > 0.025, or willingness to risk, *t*(120) = -1.07, *p* > 0.025. These findings support hypotheses 6a and 6b.

#### Affect Outcome

##### Negative affect

An analysis of covariance (ANCOVA) was conducted to test the influence of the nature of the integrity violation, response to the violation, and the influence of violator empathy on negative affect. Cynicism served as a significant covariate in the analysis. The ANCOVA revealed a main effect of response to the violation, *F*(1,231) = 6.28, *p* < 0.05, $\eta_{p}^{2}$ = 0.03, with participants in the apology condition indicating higher negative affect (*M* = 3.67, *SD* = 0.43) compared to those in the denial condition (*M* = 3.52, *SD* = 0.63). With apology eliciting higher negative affect in participants, results suggest that being aware of the boss’s culpability in the integrity violation is more negatively arousing than retaining some uncertainty regarding the matter. This finding supports hypothesis 3. We found no support, however, for hypothesis 4.

Next, the ANCOVA revealed a main effect of violator empathy, *F*(1,231) = 53.35, *p* < 0.001, $\eta_{p}^{2}$ = 0.19. Absence of violator empathy resulted in higher negative affect (*M* = 3.81, *SD* = 0.48) than its presence (*M* = 3.38, *SD* = 0.51), which is consistent with hypotheses 5c.

The ANCOVA also showed one significant two-way interaction between response to the violation and violator empathy, *F*(1,231) = 16.85, *p* < 0.001, $\eta_{p}^{2}$ = 0.07. One-tailed simple effects analyses were conducted for response to the violation at each level of violator empathy. The interaction indicated that when the violator responded with denial, absence of violator empathy resulted in higher negative affect, *t*(116) = 7.01, *p* < 0.001. When the violator apologized, absence of violator empathy resulted in higher negative affect than presence of empathy, *t*(120) = 2.54, *p* < 0.025. Thus, regardless of violator’s verbal response, absence of empathy increases one’s negative affect. Hypothesis 6C was confirmed. No evidence to support hypothesis 7c was found.

**FIGURE 1:**
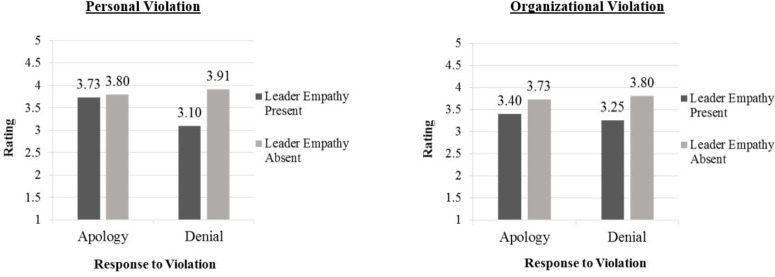
Three-way interaction between nature of the violation, response to the violation, and leader empathy on overall negative affect.

Finally, a three-way interaction was also revealed between nature of the integrity violation, response to the violation, and violator empathy, *F*(1,231) = 4.79, *p* < 0.05, $\eta_{p}^{2}$ = 0.02. Following the recommendation of Keppel (1991), separate simple two-way interactions were tested for response to the violation and violator empathy at both the personal and organizational violation levels. A significant interaction effect was revealed for response to the violation and violator empathy in the personal violation condition, *F*(1,118) = 17.22, *p* < 0.001, $\eta_{p}^{2}$ = 0.13, but not the organizational condition, *F*(1,114) = 1.89, *p* > 0.05, $\eta_{p}^{2}$ = 0.02. Thus, coupling denial with a lack of violator empathy in personal violations resulted in the highest negative affect. The nature of this interaction is portrayed in Figure 1.

## Discussion

In this study, we aimed to explore the impacts of a mistrusted party’s empathic display when coupled with two verbal responses (apology and denial) on trust repair. We also investigated whether the consequences (i.e., personal vs. organizational) of a trust violation would influence a transgressor’s ability to repair trust. Finally, we sought to confirm the nature of emotions occurring when trust is broken.

First and foremost, we were able to demonstrate that empathy plays a vital role in the trust repair process. The presence of violator empathy resulted in increased perceived integrity of the violator after an integrity violation, whereas an absence of violator empathy led to higher negative affect in participants. Our study also demonstrated that denying culpability in the transgression and coupling it with empathy led to a pronounced rise in the perceived integrity of the violator. It seems that people may be more willing to repair trust following an integrity-based violation when the violator denies responsibly and couples that response with an empathic display. According to Kim et al. (2004), denial—which explicitly proclaims accusations of guilt to be false—is beneficial to trust repair because it directs the betrayed individual to give the transgressor the benefit of the doubt. Pairing this with an empathic display on the violator’s part resulted in perceptions of higher integrity of the violator. Thus, empathy appeared to have contributed to perceived sincerity of the denial response.

In contrast to our original hypothesis, higher trust repair does not seem to occur when the consequences of a transgression are of an organizational rather than personal nature due to the potential for lesser negative affect. We did, however, find a marginally significant interactive effect of nature of the violation and response on willingness to risk. When violations carried personal ramifications, denial functioned better at repairing trust than an apology. Similarly, when violations were of a personal nature, presence of violator empathy resulted in higher perceived integrity than its absence. Although no three-way interaction was observed for these constructs, it seems that personal violations fare better in trust repair when the violator denies involvement and empathizes with the victim. While more research is necessary to ascertain the impact of the nature of the consequences on trust repair, our data suggest that this variable does not play a significant role in the process.

Another important finding of the current study was that an apology led to higher levels of negative affect in participants than a denial of guilt. This was our initial prediction based on the premise that an apology implies an admission of culpability (Kim et al., 2004) and thus may exacerbate the negative emotions felt by those harmed. Our findings also indicated that regardless of the response (i.e., denial or apology), lack of empathy resulted in higher negative affect in participants. This suggests that, if combined with a lack of empathy, denial may lead to negative affect just as much as a well-intentioned apology. This conclusion was further supported by a three-way interaction revealing that a combination of denial along with a lack of empathy during a personal violation led to the highest negative affect in wronged individuals. These findings confirm the presence of negative emotions in those harmed by trust violations and the potential for empathy to mitigate such emotions.

These findings add to our knowledge of how transgressors influence those harmed through affect, and inform emotion and trust theory. Clearly, negative emotions are invoked following transgressions, but our findings suggest that those adverse affective reactions might be intensified when the transgressor apologizes (i.e., admits culpability). Similarly, we demonstrated the effectiveness of violator empathy in repairing trust, contributing novel routes to trust repair and new elements to trust theory. More specifically, we showed that empathy contributes to the integrity trustworthiness dimension of Mayer et al. (1995)’s trust model in a positive way, suggesting that this dimension may be repaired using empathic displays from the mistrusted party. According to this trust model, replenishing the integrity trustworthiness dimension, (or any trustworthiness dimension) may ultimately contribute to trust repair. Our investigation into violator empathy suggests that the role of affect in trust is ripe for future exploration. We hope future researchers continue to enrich trust theory with further consideration of the role of emotion in this process.

Practically, there are several important implications of this research. Individuals harmed by trust violations should be aware that there are transgressors who may be accountable yet refuting their involvement and using empathy in order to deceive an unsuspecting person into unwarranted trust repair. By making individuals aware of this possibility, we hope that they will become more cautious when granting trust to alleged violators. For violators who are genuinely hoping to gain the benefit of the doubt or delay an unjustly negative conclusion, our findings would suggest not only denying guilt, but uniting that response with a genuine empathic display. Doing so would likely result in a higher probability of repairing trust following the resolution of the matter and suspend judgment in the interim. Additionally, in the case of violations concerning matters of integrity, it appears that apologizing is not only an inferior response but may also exacerbate negative affective reactions in harmed individuals. Violators may wish to be cautious when deploying an apologetic response due to the unintended yet probable negative effect it seems to have on harmed individuals’ emotional reactions.

### Limitations and Directions for Future Research

There are several limitations to this study that must be noted. One limitation is the use of low-fidelity or scenario-based simulations to describe the violation to the participants and manipulate the variables of interest. Though low-fidelity approaches are, by definition, not perfectly real, meta-analyses have shown this technique to be reliable and valid (McDaniel et al., 2001; Christian et al., 2010). Additionally, low-fidelity simulations are commonly used in employment settings as selection tools to measure domain-specific knowledge and skill, and are touted for their cost effectiveness (Motowidlo et al., 1990; Lievens and Patterson, 2011). Nevertheless, researchers may wish to replicate this work using a simulation of higher fidelity. Recording videos of transgressors displaying empathy visually may lend further fidelity to the affective portion of the study and promote emotional contagion (Hatfield et al., 1994), which may, in turn, increase the influence of the emotional display. However, researchers using this option will need to take the necessary steps to eliminate extraneous variance introduced by violator attractiveness, voice, or other irrelevant cues which may otherwise distort the true findings.

Along similar lines, another limitation is the laboratory setting of our study, which may decrease the external validity of the findings due to a potential disconnect between experiencing hypothetical versus realized integrity violations. The laboratory nature of this study has its merits, however, as it allowed us to isolate the variables of interest and study them without concern for irrelevant cues rife in field settings, thereby strengthening the study’s internal validity. Moreover, participants reported being highly engaged in the study as indicated by very high scores on the post-survey (*M* = 6.16, *SD* = 0.98). Researchers still concerned about this method are encouraged to conduct similar work in more realistic settings.

An additional limitation involves our sample, which consisted of fairly young individuals with limited work and life experiences. As mentioned earlier, participants were on average 19.28 years of age (*SD* = 1.41; range = 18–32 years) and possessed an average of 2.4 years of work experience. Extant research has indicated that younger, as opposed to midlife and older adults, are less likely to forgive (Toussaint et al., 2001), while other studies have revealed a positive linear relationship between age and forgiveness (Girard and Mullet, 1997). Given this trend in the forgiveness literature, it appears that our study only garnered responses from young adults. Although we controlled statistically for age in this study, future researchers may consider extending our work to include responses from midlife and older adults to generate a more complete picture. Similar considerations should be made in reference to work experience.

Yet another limitation involves the lack of measurement of trust over time in this study. As clarified by Lewicki and Brinsfield (2017), trust fluctuates over time and is a fairly multifaceted construct. Therefore, we recommend for future researchers to take into account the dynamic nature of trust and measure trust at multiple points in time, thus providing a more comprehensive picture of the trust repair cycle. Along similar lines, measuring initial trust (i.e., trust prior to the violation) would allow for the ultimate gauging of the trust repair strategy under investigation.

We also urge researchers to consider varying levels of severity of integrity violations in any forthcoming investigations. Grover et al. (2014), for instance, provided a taxonomy of recoverable and irrecoverable trust violations, ultimately specifying that severity of the breach is an important consideration. Specifically, the authors showed that regardless of the type of violation (i.e., competence-, integrity-, or benevolence-based), trust was reparable, but only if the violation was not too severe. Severe integrity violations were categorized under irrecoverable trust violations. More recently, Grover et al. (2017) further indicated that severity of a violation carries much weight when it comes to restoring trust in leader–follower relationships. Ultimately, as Krylova et al. (2017) recently stated in their review of literature on the subject, “…not all integrity-based transgressions are created equal” (p. 199), so considerations of severity, intentionality, and methods of delivery of responses all matter.

Another potential arena for future investigation is within the cross-cultural domain. Specifically, we recognize that trust repair might be culturally bound, especially across western and eastern countries. Methods for trust repair that may show promise in individualistic cultures, for instance, may not be well received in collectivistic cultures. Future work should consider how culture limits or promotes trust repair efforts.

Finally, we only considered one type of affective display in this study and fixed the gender of the violator to be male across all conditions. Some work exists suggesting that female and male leaders are rated differently with respect to effectiveness when displaying gender-stereotypical emotions. A study by Lewis (2000) showed that female leaders displaying anger and sadness were rated to be less effective than when they displayed no emotion. On the other hand, male leaders were rated similarly regardless of whether they displayed no emotion or anger (a gender stereotypical emotion). It may be of interest to researchers of the trust construct to vary the gender of the violator and couple it with particular affective displays to investigate whether the consequences of those emotional displays vary based on the gender of the transgressor. Furthermore, one can elucidate the usefulness of those emotional displays when united with particular verbal responses. For instance, does observing a negative emotional display combined with denial from a leader promote negative affect in followers and thus negate trust repair normally observed of the denial response to an integrity violation in the literature? Alternatively, gender of the harmed party may also influence willingness to forgive (Miller et al., 2008) and the manner by which the stressful situation such as an integrity breach is dealt with (Taylor et al., 2000). Specifically, Taylor et al. (2000) discussed a tendency to tend-and-befriend on the part of women when coping with stress, in contrast to the fight-or-flight response commonly preferred by men. This suggests that women may draw on the perspective of their social network prior to making the decision to reconcile or trust the transgressor in the future. Moreover, the tenets of tend-and-befriend indicate that women are socialized to forgive transgressions as a means to cope with the associated stress. Meta-analytic evidence supports this proposition demonstrating the more forgiving nature of females versus males (Miller et al., 2008). Future research possibilities are rich in this area and could contribute to our understanding of trust, leadership, and the role of emotions in the workplace.

## Ethics Statement

This study was carried out in accordance with the recommendations of the University of Oklahoma, Institutional Review Board. The protocol was approved by the University’s Institutional Review Board. All subjects gave written informed consent in accordance with the Declaration of Helsinki.

## Author Contributions

All authors listed have made a substantial, direct and intellectual contribution to the work, and approved it for publication.

## Conflict of Interest Statement

The authors declare that the research was conducted in the absence of any commercial or financial relationships that could be construed as a potential conflict of interest.
