# Supplementary material for: Denial and Empathy: Partners in Employee Trust Repair?
Source: Front Psychol. 2019 Jan 22;10:19. doi: 10.3389/fpsyg.2019.00019 (PMC6349725; doi:10.3389/fpsyg.2019.00019)
Supplement: Supplementary file 1 [file Data_Sheet_1.docx]

Appendix A: Sample Manipulation

**Organizational Violation, Denial, Violator Empathy Absent**

Legend:

Single underline = organizational violation

Double underline = no violator empathy

Italics = *denial*

You are member of a large accounting firm for a well-known electronics company. You have worked for this firm for 2 years, giving it your all. Your job entails keeping track of the company’s earnings and expenditures. You are then responsible for delivering these documents to the investors so that they are aware of how the company is doing. However, because your direct manager, Chris Johnson, is a new member of the team, he insists that all documents go through him for inspection and that he will then send them out. He explains to you that this will only be done for a brief period of time so that he can gain a better understanding of the company as a whole. He also clarifies that this is his way of “staying in the loop” when it comes to company finances.

At the end of the fiscal year, one of the investment firms contacts you requesting access to company’s financial records, which is their right as a shareholder. Since this is something that occurs often, you gather all the requested information and send it to the investor without further thought. A few weeks later, you are summoned into a meeting with the company’s chief financial officer (CFO). You are shocked when the CFO informs you that the numbers you provided the investors were incorrect, underreporting company expenditures, and overstating the company’s profits, making the company appear to be doing much better than it is. Due to this, the investors are threatening to pull their interest from the company. In order to restore investor confidence, the company has decided to withhold employee bonuses this year to close the margin of error between reported and actual profits. You try to explain to the CFO that your reports were accurate and that as a matter of fact, your manager, Chris Johnson, double-checked everything prior to sending it out. The CFO is not interested in your excuses, informing you that as your signatures are on the documents it is your responsibility to ensure that they are correct.

You leave the meeting angry, disheartened, and confused. You just cannot understand how you could have made such a mistake. After double-checking your original reports, you are convinced that Chris Johnson is somehow responsible for the inaccuracies and you decide to confront him. You find him and relate all that occurred in your meeting with the CFO and show that the documents the CFO provided do not match your original reports. You then ask Chris Johnson directly if he had anything to do with the documents being changed. *Chris Johnson then states the following: “I had nothing to do with the inaccuracies in the reports; I’m not sure why you would think that I did.* I hope that you can find a way to make the best out of this situation since we must continue working together.” As he utters these words, you find that his tone of voice lacks true compassion and understanding of your feelings.

**Organizational Violation, Denial, Violator Empathy Present**

Legend:

Single underline = organizational violation

Double underline = violator empathy

Italics = *denial*

You are member of a large accounting firm for a well-known electronics company. You have worked for this firm for 2 years, giving it your all. Your job entails keeping track of the company’s earnings and expenditures. You are then responsible for delivering these documents to the investors so that they are aware of how the company is doing. However, because your direct manager, Chris Johnson, is a new member of the team, he insists that all documents go through him for inspection and that he will then send them out. He explains to you that this will only be done for a brief period of time so that he can gain a better understanding of the company as a whole. He also clarifies that this is his way of “staying in the loop” when it comes to company finances.

At the end of the fiscal year, one of the investment firms contacts you requesting access to company’s financial records, which is their right as a shareholder. Since this is something that occurs often, you gather all the requested information and send it to the investor without further thought. A few weeks later, you are summoned into a meeting with the company’s chief financial officer (CFO). You are shocked when the CFO informs you that the numbers you provided the investors were incorrect, underreporting company expenditures, and overstating the company’s profits, making the company appear to be doing much better than it is. Due to this, the investors are threatening to pull their interest from the company. In order to restore investor confidence, the company has decided to withhold employee bonuses this year to close the margin of error between reported and actual profits. You try to explain to the CFO that your reports were accurate and that as a matter of fact, your manager, Chris Johnson, double-checked everything prior to sending it out. The CFO is not interested in your excuses, informing you that as your signatures are on the documents it is your responsibility to ensure that they are correct.

You leave the meeting angry, disheartened, and confused. You just cannot understand how you could have made such a mistake. After double-checking your original reports, you are convinced that Chris Johnson is somehow responsible for the inaccuracies and you decide to confront him. You find him and relate all that occurred in your meeting with the CFO and show that the documents the CFO provided do not match your original reports. You then ask Chris Johnson directly if he had anything to do with the documents being changed. Chris Johnson hears you out, all the time gazing at you with concern, and then states the following: *“I had nothing to do with the inaccuracies in the reports; I’m not sure why you would think that I did.* I understand that you are angry and confused after the day you’ve had and wish there was something more I could do. I hope that you can find a way to make the best out of this situation since we must continue working together. I know you are very capable and I’m sure this will all blow over eventually.” As he utters these words, you believe that he really understands how you are feeling.

Appendix B: Trust Beliefs Scale

Please choose the one number for each statement that comes closest to reflecting your opinion about it:

1 = Strongly disagree

2 = Moderately disagree

3 = Slightly disagree

4 = Neither disagree nor agree

5 = Slightly agree

6 = Moderately agree

7 = Strongly agree

| ______ | 1. I like Chris Johnson’s values. |
| --- | --- |
| ______ | 1. Sound principles seem to guide Chris Johnson’s behavior. |
| ______ | 1. Chris Johnson has a great deal of integrity. |
|  |  |

Appendix C: Trusting Intentions Scale

Please choose the one number for each statement that comes closest to reflecting your opinion about it:

1 = Strongly disagree

2 = Moderately disagree

3 = Slightly disagree

4 = Neither disagree nor agree

5 = Slightly agree

6 = Moderately agree

7 = Strongly agree

| ______ | 1. I wouldn’t let Chris Johnson have any influence over issues that are important to me. |
| --- | --- |
| ______ | 1. I would keep an eye on Chris Johnson. |
| ______ | 1. I would give Chris Johnson a task or problem that was critical to me, even if I could not monitor his actions. |
|  |  |

Appendix D: Negative Affect Questions

Now that you have read the case, please answer these questions to the best of your ability.

1. Describe your feelings about your boss’ actions. ________________________________________________________________________________________________________________________________________________________________________________________________________________________________________________________________________________________________________________________________________________________________________________________________________________________________________________________________________
2. Describe your feelings regarding the meeting you just had with your boss during which you confronted him about his actions.

________________________________________________________________________________________________________________________________________________________________________________________________________________________________________________________________________________________________________________________________________________________________________________________________________________________________________________________________________________________________________________________________________________________________________________________________________________________________
